# Optimality condition and iterative thresholding algorithm for $l_p$-regularization problems

**DOI:** 10.1186/s40064-016-3516-3

**Published:** 2016-10-26

**Authors:** Hongwei Jiao, Yongqiang Chen, Jingben Yin

**Affiliations:** 1 School of Mathematical Sciences, Henan Institute of Science and Technology, Xinxiang, 453003 China; 2 College of Mathematics and Information Science, Henan Normal University, Xinxiang, 453007 China

**Keywords:** Optimality condition, $l_p$-regularization problems, Iterative thresholding algorithm, Global optimum solution, Fixed point, 90C26, 90C46, 90C90

## Abstract

This paper investigates the $l_p$-regularization problems, which has a broad applications in compressive sensing, variable selection problems and sparse least squares fitting for high dimensional data. We derive the exact lower bounds for the absolute value of nonzero entries in each global optimal solution of the model, which clearly demonstrates the relation between the sparsity of the optimum solution and the choice of the regularization parameter and norm. We also establish the necessary condition for global optimum solutions of $l_p$-regularization problems, i.e., the global optimum solutions are fixed points of a vector thresholding operator. In addition, by selecting parameters carefully, a global minimizer which will have certain desired sparsity can be obtained. Finally, an iterative thresholding algorithm is designed for solving the $l_p$-regularization problems, and any accumulation point of the sequence generated by the designed algorithm is convergent to a fixed point of the vector thresholding operator.

## Background

In this paper, we investigate the following $l_p$-regularization problems

1 $$\begin{aligned} \min _{s\in {R}^n} f_\lambda (s):= \Vert As-b \Vert _2^2+ \lambda \Vert s \Vert _p^p \end{aligned}$$

where $A\in {R}^{m\times n},~b\in {R}^m,~\lambda \in (0, \infty ), \Vert s \Vert _p^p=\sum \nolimits _{i=1}^{n} |s_i |^p, ~p\in (0, 1)$. The problem (1) has a broad applications in compressive sensing, variable selection problems and sparse least squares fitting for high dimensional data (see Chartrand and Staneva 2008; Fan and Li 2001; Foucart and Lai 2009; Frank and Freidman 1993; Ge et al. 2011; Huang et al. 2008; Knight and Wu 2000; Lai and Wang 2011; Natarajan 1995). The objective function of the problem (1) is consisted by a data fitting term $\Vert As-b \Vert _2^2$ and a regularization term $\lambda \Vert s \Vert _p^p$. In Chen et al. (2014) point out that the $l_2$-$l_p$ minimization problem (1) is a strongly NP-hard problem. Comparing with using the $l_1$ norm, using the $l_p$ quasi-norm in the regularization term we can find sparser solution, which has been extensively discussed in Candès et al. (2008), Chartrand (2007a, b), Chartrand and Yin (2008), Chen et al. (2010), Tian and Huang (2013), Tian and Jiao (2015), Xu et al. (2010, 2012), Shehu et al. (2013, 2015), Bredies et al. (2015), Fan et al. (2016). In Chen et al. (2010), Chen et al. derive the lower bounds for the absolute value of nonzero entries in each local optimum solution of the model. Xu et al. (2012) presented an analytical expression in a thresholding form for the resolvent of gradient of $\Vert s\Vert _{1/2}^{1/2}$ and developed an alternative feature theorem on optimum solutions of the $L_{1/2}$ regularization problem, and proposed an iterative half thresholding algorithm for fast solving the problem. But there is no result for the characteristics of the global optimum solution for the problem (1).

In this article, we pay more attention to derive the characteristics of the global optimum solution of problem (1), which is inspired by Xu et al. (2012). The remaining sections of the paper are organized as follows. In “Technical preliminaries” section, we portray some important technical results. “Lower bound and optimality conditions” section first develop the proximal operator associated with a non-convex $l_p$ quasi-norm, which can be looked as an extension of the well-known proximal operator associated with convex functions. Next, an exact lower bound for the absolute value of nonzero entries in every global optimum solution of (1) is derived, which clearly demonstrates the relation between the sparsity of the optimum solution and the choice of the regularization parameter and norm. We also establish the necessary condition for global optimum solutions of the $l_p$-regularization problems, i.e., the global optimum solutions are fixed points of a vector thresholding operator. In “Choosing the parameter λ for sparsity” section, we also propose a sufficient condition on the selection of $\lambda$ to meet the sparsity requirement of global minimizers of the $l_p$-regularization problems. “Iterative thresholding algorithm and its convergence” section proposes an iterative thresholding algorithm for the $l_p$-regularization problems, and any accumulation point of the sequence produced by the designed algorithm is convergent to a fixed point of the vector thresholding operator. Finally, some conclusions are drawn in “Numerical experiments” section.

## Technical preliminaries

By utilizing the objective function’s separability and the operator splitting technique, the $l_p$-regularization problems (1) can be converted into *n* homologous single variable minimization problems defined on $(-\infty , +\infty )$. Therefore, at first we investigate the homologous single variable minimization problem

2 $$\begin{aligned} \min _{s\in {R}}~g_r(s):=s^2-2rs+\lambda |s|^{p}, \end{aligned}$$

where $\lambda >0$ and $p\in (0,1)$ are all any real numbers, $s\in {R}$ is a variable and $r\in {R}$ is a parameter. Besides, we only need to consider the following two sub-problems

3 $$\begin{aligned} \min _{s\ge 0}~g_r(s)& = s^2-2rs+\lambda s^{p}, \end{aligned}$$

4 $$\begin{aligned} \min _{s\le 0}~g_r(s)& = s^2-2rs+\lambda (-s)^{p}. \end{aligned}$$

In Chen et al. (2014), investigated the subproblem (3) and presented some results, which can be used to derive our conclusions. Let

5 $$\begin{aligned} {\bar{r}}\, {:=} \,\frac{2-p}{1-p}[ \lambda p(1-p)/2 ]^{1/(2-p)}>0, \end{aligned}$$

6 $$\begin{aligned} {\bar{s}} \;{:=} \;[ \lambda p(1-p)/2 ]^{1/(2-p)}>0. \end{aligned}$$

### **Lemma 1**

(Lemma.2.2, Chen et al. 2014) *For any* $s>0$, *denote* $G(s,r) := [g_r(s)]^{'}=2s-2r+\lambda ps^{p-1}$. *For any known* $r_0>{\bar{r}}$, set $s_0 ~( s_0>{\bar{s}})$ *be the positive root of the equation* $G(s,r_0)=0$, *where* ${\bar{r}}$ *and* ${\bar{s}}$ *are given in* (5) *and* (6). *Then, there is a unique implicit function* $s=h_{\lambda , p}(r)$ *define on* $({\bar{r}}, +\infty )$, *which satisfies* $s_0=h_{\lambda , p}(r_0)$, $h_{\lambda , p}(r)>{\bar{s}}$ *and* $G(h_{\lambda , p}(r), r)\equiv 0$ *for* $\forall r\in ({\bar{r}}, +\infty )$. *Furthermore, for the function* $s=h_{\lambda , p}(r)$, *the following conclusions hold*:

1. $s=h_{\lambda , p}(r)$ *is a continuous function defined on* $({\bar{r}}, +\infty )$.
2. $s=h_{\lambda , p}(r)$ *is a differentiable function over* $({\bar{r}}, +\infty )$ and $h_{\lambda , p}^{'}(r)=\frac{2}{2+\lambda p(p-1)h_{\lambda , p}^{p-2}(r)}$.
3. $s=h_{\lambda , p}(r)$ *is a strictly increasing function over* $({\bar{r}}, +\infty )$.

*Moreover, if* $r>{\bar{r}}$, *then* $s=h_{\lambda , p}(r)$ *is the sole local minimizer of* $g_r(s)$ *over* $(0, +\infty )$.

### **Lemma 2**

(Prop.2.4, Chen et al. 2014) *Set* $s^*$ *be the global optimum solution for the problem* (3), *then we have*

$$\begin{aligned} s^*=h_\lambda (r):=\left\{ \begin{array}{lll} h_{\lambda , p}(r) , & r>r^*\\ (\lambda (1-p) )^{1/(2-p)} \quad \mathrm{or}\; 0, & r=r^* \\ 0, & r<r^* \end{array} \right. \end{aligned}$$

*where* $r^*:=\frac{2-p}{2(1-p)} [\lambda (1-p) ]^{1/(2-p)}$, $h_{\lambda , p}(r)$ *is defined by Lemma 1*.

### **Proposition 1**

*Set* $s^*$ *be the global optimum solution for the problem *(2), *then we have*

7 $$\begin{aligned} s^*=h_\lambda (r):=\left\{ \begin{array}{lllll} h_{\lambda , p}(r) , & \quad r>r^* \\ L \quad \mathrm{or}\; 0, & \quad r=r^*\\ 0, & \quad -r^*<r<r^* \\ -L \quad \mathrm{or}\; 0, & \quad r=-r^*\\ -h_{\lambda , p}(-r) , & \quad r<-r^* \end{array} \right. \end{aligned}$$

*where* $r^*:=\frac{2-p}{2(1-p)} [\lambda (1-p) ]^{1/(2-p)}$, $h_{\lambda , p}(r)$ *is defined in Lemma 1* *and* $L:=(\lambda (1-p) )^{1/(2-p)}$.

### *Proof*

If $s\ge 0$, then $g_r(s)=s^2-2rs+\lambda s^{p}$. Let $s^*_1$ is a global optimum solution for the problem (3), then from Lemma 2, we have

8 $$\begin{aligned} s^*_1=h_\lambda (r)=\left\{ \begin{array}{lll} h_{\lambda , p}(r) , & \quad r>r^*\\ (\lambda (1-p) )^{1/(2-p)} \quad \mathrm{or} \; 0, & \quad r=r^*\\ 0. & \quad r<r^* \end{array} \right. \end{aligned}$$

If $s\le 0$, then $g_r(s)=s^2-2rs+\lambda (-s)^{p}=(-s)^2+2r(-s)+\lambda (-s)^{p}$. Let $y=-s$, we have $y\ge 0$ and $g_{(-r)}(y)=y^2+2ry+\lambda y^p$, we follow the first case. If $y^*$ is a global optimum solution for the problem $g_{(-r)}(y)$ over $[0, +\infty )$, then from Lemma 2, we have

$$\begin{aligned} y^*=h_\lambda (-r)=\left\{ \begin{array}{lll} h_{\lambda , p}(-r) , & \quad -r>r^* \\ (\lambda (1-p) )^{1/(2-p)} \quad \mathrm{or}\; 0, & \quad -r=r^*\\ 0. & \quad -r<r^* \end{array} \right. \end{aligned}$$

Therefore, if $s\le 0, ~s^*_2$ is a global optimum solution for the problem $\min _{s\in {R}_-}~g_r(s)=s^2-2rs+\lambda (-s)^{p}$, then we have

9 $$\begin{aligned} s^*_2=-y^*=\left\{ \begin{array}{lll} -h_{\lambda , p}(-r) , & \quad r<-r^* \\ -(\lambda (1-p) )^{1/(2-p)} \quad \mathrm{or} \; 0, & \quad r=-r^*\\ 0. & \quad r>-r^* \end{array} \right. \end{aligned}$$

Combining (8) and (9) together, we can get (7). Therefore, the proof is complete. $\square$

### **Proposition 2**

*Assume that* $s^*$ *is a global optimum solution for the problem* (2). *When* $|r|=r^*$ *given in Proposition 1*, *set* $s^*=h_{\lambda }(r)$ *be simultaneously zero or nonzero. Then the following conclusions hold:*

1. *The function* $h_{\lambda }(r)$ *is an odd function over* $(-\infty , +\infty )$.
2. *The function* $h_{\lambda }(r)$ *is continuous over* $(r^*, +\infty )$, *furthermore*, $\lim _{r\downarrow r^*} h_{\lambda }(r)=L$.
3. *The function* $h_{\lambda }(r)$ *is differentiable over* $(r^*, +\infty )$.
4. *The function* $h_{\lambda }(r)$ *is strictly increasing over* $(r^*, +\infty )$.

### *Proof*

By Proposition 1 and Lemma 1, this proposition can be followed. $\square$

When $p=1/2$, in Xu et al. (2012), $h_{\nu , p}(r)$ of (7) has the following analytic corollary.

### **Corollary 1**

(Theo. 1, Lemm. 1 and 2, Xu et al. 2012) *When* $p=1/2$, *the global optimum solution* $s^*$ *of problem* (2) *has the following results*:

10 $$\begin{aligned} s^*=h_\lambda (r):=\left\{ \begin{array}{lllll} h_{\lambda , 1/2}(r), & \quad r>r^*\\ (\lambda /2)^{2/3} \quad \mathrm{or} \; 0, & \quad r=r^*\\ 0, & \quad -r^*<r<r^* \\ -(\lambda /2)^{2/3} \quad \mathrm{or}\; 0, & \quad r=-r^*\\ -h_{\lambda , 1/2}(-r) , & \quad r<-r^* \end{array} \right. \end{aligned}$$

*where* $$h_{\lambda , 1/2}(r) =\frac{2}{3}r(1+\mathrm {cos}(\frac{2\pi }{3}-\frac{2\varphi (r)}{3}))$$, $$\varphi (r)=\mathrm {arccos}(\frac{\lambda }{8}(\frac{|r|}{3} )^{-3/2} )$$ *and* $$r^* =\frac{\root 3 \of {54}}{4}\lambda ^{2/3}$$.

### *Proof*

A brief proof is presented here for completeness. When $p=1/2$, we have $r^* =\frac{\root 3 \of {54}}{4}\lambda ^{2/3}$. When $|r|>r^*$, $s^*=h_{\lambda }(r)\ne 0$, by Proposition 2, then $h_{\lambda }(r)$ is the root of the equation

$$\begin{aligned} s-r+\frac{\lambda \mathrm{sign(s)}}{4 \sqrt{|s|}}=0, \end{aligned}$$

which is followed by the first order optimum condition of (2). By Theorem 1 of Xu et al. (2012), we have $h_{\lambda , 1/2}(r) =\frac{2}{3}r(1+\mathrm {cos}(\frac{2\pi }{3}-\frac{2\varphi (r)}{3})),~ \varphi (r)=\mathrm {arccos}(\frac{\lambda }{8}(\frac{|r|}{3} )^{-3/2} )$. The proof is completed. $\square$

## Lower bound and optimality conditions

In this section, by using function’s separability and the operator splitting technique, we propose the proximal operator associated with $l_p$ quasi-norm. Next, we present the properties of the global optimum solutions of the $l_p$-regularization problems (1). For convenience, first of all, we define the following thresholding function and thresholding operators.

### **Definition 1**

($\mathrm {p}$ *thresholding function*) Assume that $r\in {R}$, for any $\lambda > 0$, the function $h_\lambda (r)$ defined in (7) is called as a $\mathrm {p}$ thresholding function.

### **Definition 2**

(*Vector* $\mathrm {p}$ *thresholding operator*) Assume that $s\in {R}^n$, for any $\lambda > 0$, the vector $\mathrm {p}$ thresholding operator $H_\lambda (s)$ is defined as

$$\begin{aligned} H_\lambda (s):=(h_\lambda (s_1),h_\lambda (s_2),\ldots ,h_\lambda (s_n))^T. \end{aligned}$$

In this section, one of the main results is a proximal operator associated with the non-convex $l_p~(0<p<1)$ quasi-norm, and which can be also looked as an extension of the well-known proximal operator associated with convex functions.

### **Theorem 1**

*For given a vector* $y\in {R}^n$ *and constants* $\lambda >0, ~0<p<1$. *Assume that* $s^*$ *be the global optimum solution of the following problem*

11 $$\begin{aligned} \min _{s\in {R}^n}~f(s):=\Vert s-y \Vert _2^2+\lambda \Vert s \Vert _{p}^{p}, \end{aligned}$$

then $s^*$ can be expressed as

$$\begin{aligned} s^*=H_{\lambda }(y). \end{aligned}$$

*Furthermore, we can get the exact number of global optimum solutions for the problem.*

### *Proof*

From

$$\begin{aligned} f(s)&=\Vert s-y \Vert _2^2+\lambda \Vert s \Vert _{p}^{p}=\Vert s \Vert _2^2-2\langle s, y \rangle +\Vert y \Vert _2^2 +\lambda \Vert s \Vert _{p}^{p} \\&=\sum \limits _{i=1}^{n} \left( s^2_i- 2y_i s_i+\lambda {|s_i|}^p \right) +\Vert y \Vert _2^2. \end{aligned}$$

Let $$g_{y_i}(s_i)=s^2_i- 2y_i s_i+\lambda {|s_i|}^p$$, then

$$\begin{aligned} f(s)=\sum \limits _{i=1}^{n} g_{y_i}(s_i)+\Vert y \Vert _2^2. \end{aligned}$$

Therefore, to solve the problem (11) is equivalent to solving the following *n* problems, for each $i=1,2,\ldots ,n$,

12 $$\begin{aligned} \min _{s_i \in {R}}~ g_{y_i}(s_i). \end{aligned}$$

By Proposition 1, for each $i=1,2,\ldots ,n$, we can follow

$$\begin{aligned} s_i^*=\arg \min_{s_i \in {R}}~ g_{y_i}(s_i)=h_{\lambda }(y_i), \end{aligned}$$

and if $|y_i |=r^*:=\frac{2-p}{2(1-p)} [\lambda (1-p) ]^{1/(2-p)}$, the problem (12) has two solutions; else, unique solution. Hence we can know the exact number of global optimum solutions of (11). The proof is thus complete. $\square$

For any $\lambda ,~ \mu > 0,~ 0<p<1,$ and $z\in {R}^n$, let

13 $$\begin{aligned} f_{\mu }(s,z):=\mu (f_{\lambda }(s)-\Vert As-Az \Vert _2^2)+ \Vert s-z \Vert _2^2, \end{aligned}$$

For simplicity, let

14 $$\begin{aligned} B_{\mu }(z):=z+{\mu } A^T(b-Az). \end{aligned}$$

### **Theorem 2**

*Assume that* $s^*\in {R}^n$ *be the global minimizer of* $f_\mu (s,z)$ *for any fixed* $\lambda>0, \mu >0$ *and* $z\in {R}^n$, *then we have*

15 $$\begin{aligned} s^*=H_{\lambda \mu }(B_{\mu }(z)). \end{aligned}$$

### *Proof*

Without loss of generality, $f_\mu (s,z)$ can be rewritten as

$$\begin{aligned} \begin{array}{lll} f_\mu (s,z)&=&\mu (\Vert As-b \Vert _2^2+\lambda \Vert s \Vert _p^p -\Vert As-Az \Vert _2^2)+ \Vert s-z \Vert _2^2\\ &=&\lambda \mu \Vert s \Vert _p^p+\Vert s \Vert _2^2-2\langle s, z+\mu A^T (b-Az) \rangle +\Vert z \Vert _2^2+\mu \Vert b \Vert _2^2-\mu \Vert Az \Vert _2^2\\ &=&\Vert s -B_\mu (z)\Vert _2^2+\lambda \mu \Vert s \Vert _p^p+ \Vert z \Vert _2^2+\mu \Vert b \Vert _2^2-\mu \Vert Az \Vert _2^2-\Vert B_\mu (z) \Vert _2^2. \end{array} \end{aligned}$$

Therefore, to solve $\min _{s\in {R}^n} f_\mu (s,z)$ for any fixed $\nu , \mu$ and *Y* is equivalent to solving

$$\begin{aligned} \min _{s\in {R}^n}\large \{\Vert s-B_\mu (z) \Vert _2^2+\lambda \mu \Vert s \Vert _p^p \}. \end{aligned}$$

By Theorem 1, thus the proof is complete. $\square$

### **Lemma 3**

*If* $s^*\in {R}^n$ *is a global minimizer of the problem* (1) *for any fixed* $\nu >0$ *and for any fixed* $\mu$ *which satisfies* $0 <\mu \le \Vert A \Vert ^{-2}$, *then* $s^*$ *is also a global minimizer of* $f_\mu (s, s^*)$, *that is*,

$$\begin{aligned} f_{\mu }(s^*, s^*)\le f_{\mu }(s, s^*) \quad \mathrm{for\;all}\;s\;\in {R}^{n}. \end{aligned}$$

### *Proof*

For any $s\in {R}^{n}$, Since $0 <\mu \le \Vert A \Vert ^{-2}$, we have

$$\begin{aligned} \Vert s-s^* \Vert _2^2-\mu \Vert As-As^* \Vert _2^2\ge \Vert s-s^* \Vert _2^2-\mu \Vert A \Vert ^{2} \Vert s-s^* \Vert _2^2\ge 0. \end{aligned}$$

Hence,

$$\begin{aligned} \begin{array}{lll} f_{\mu }(s, s^*)&=&\mu (f_\lambda (s)-\Vert As-As^* \Vert _2^2)+ \Vert s-s^* \Vert _2^2\\ &=&\mu (\Vert As-b \Vert _2^2+\lambda \Vert s \Vert _p^p)+ (\Vert s-s^* \Vert _2^2-\mu \Vert As-As^* \Vert _2^2)\\ &\ge & \mu (\Vert As-b \Vert _2^2+\lambda \Vert s \Vert _p^p) \\ &=& \mu f_\lambda (s)\ge \mu f_\lambda (s^*) \\ &=& f_{\mu }(s^*, s^*) \\ \end{array} \end{aligned}$$

the proof is complete. $\square$

### **Theorem 3**

*For any given* $\lambda >0,~0<\mu \le \Vert A \Vert ^{-2}$, *if* $s^*$ *be the global optimum solution of the problem* (1), *then* $s^*$ satisfies

16 $$\begin{aligned} s^*=H_{\lambda \mu }(B_\mu (s^*)). \end{aligned}$$

*Especially, we have*

17 $$\begin{aligned} \begin{array}{ll} s^*_i & =h_{\lambda \mu }([B_\mu (s^*)]_i)\\ & =\left\{ \begin{array}{llll} h_{\lambda \mu , p}([B_\mu (s^*)]_i), & \quad \mathrm{if} \quad {[B_\mu (s^*)]}_i> r^* \\ L~\mathrm{or}~0, & \quad \mathrm{if} \quad {[B_\mu (s^*)]}_i= r^* \\ 0, & \quad \mathrm{if} \quad -r^*<{[B_\mu (s^*)]}_i< r^* \\ -L~\mathrm{or}~0, & \quad \mathrm{if} \quad {[B_\mu (s^*)]}_i= -r^* \\ -h_{\lambda \mu , p}(-[B_\mu (s^*)]_i), & \quad \mathrm{if} \quad {[B_\mu (s^*)]}_i< -r^* \end{array} \right. \end{array} \end{aligned}$$

*where* $$r^*:=\frac{2-p}{2(1-p)} [\lambda \mu (1-p) ]^{1/(2-p)}$$ *and* $$L:=(\lambda \mu (1-p) )^{1/(2-p)}$$.

*Furthermore, we have: if* $s^*_i\in (-L, L)$, *then* $s^*_i=0$.

### *Proof*

Since $s^*$ is a global minimizer of $f_\mu (s, z)$ for given $z=s^*$, by Theorem 2 and Lemma 3, we can directly get (16) and (17). By proposition 2, we can follow that

$$\begin{aligned} \lim _{r\downarrow r^*} h_{\lambda \mu }(r)=[\lambda \mu (1-p)]^{\frac{1}{2-p}} \;{=:}\;L. \end{aligned}$$

By Proposition 2, combining with the strict monotonicity of $h_{\lambda \mu }(\cdot )$ on $({\bar{r}}, +\infty )$ and $(-\infty , -{\bar{r}})$, we can follow that $s^*_i> L$ as ${[B_\mu (s^*)]}_i> r^*$, $s^*_i< -L$ as ${[B_\mu (s^*)]}_i< -r^*$ and $|s^*_i|=L$ as $| {[B_\mu (s^*)]}_i| = r^*$. Therefore, the proof is completed. $\square$

### *Remark 1*

In Theorem 3, the necessary condition for global optimum solutions of the $l_p$-regularization problems is established, which is a thresholding expression associated with the global optimum solutions. Particularly, the global optimum solutions for the problem (1) are the fixed points of a vector-valued thresholding operator. In contrast, the conclusion does not hold in general, i.e., a point satisfying (16) is not the global optimum solution for the $l_p$-regularization problems (1) in general. This is related to the nature of the matrix *A*, for an instance, when $A\equiv I$ and $\mu =1$, a fixed point of (16) is the global optimum solution for the $l_p$-regularization problems (1) (i.e., Theorem 1).

### *Remark 2*

In Theorem 3, the exact lower bound for the absolute value of nonzero entries in every global optimum solution of the model is also provided, which can be used to identify zero entries precisely in any global optimum solution. These lower bounds clearly demonstrate the relationship between the sparsity of the global optimum solution and the choices of the regularization parameter and norm, therefore, our theorem can be used to select the desiring model parameters and norms.

## Choosing the parameter $\lambda$ for sparsity

In many applications such that sparse solution reconstruction and variable selection, one need to seek out least square estimators with no more than *k* nonzero entries. Chen et al. (2014) present a sufficient condition on $\lambda$ for global minimizers of the $l_p$-regularization problems, which have desirable sparsity, and which are based on the lower bound theory in local optimum solutions. In this paper, we also present a sufficient condition on $\lambda$ for global minimizers of the $l_p$-regularization problems, which also have desirable sparsity, but which are based on the lower bound theory in global optimum solutions.

### **Theorem 4**

*Set*

18 $$\begin{aligned} \beta (k)=k^{(p-2)/2} [\mu (1-p)]^{-p/2}\Vert b \Vert ^{2-p}, \quad 1\le k \le n. \end{aligned}$$

*The following conclusions hold.*

1. *If* $\lambda \ge \beta (k)$, *then any global minimizer* $s^*$ *of the* $l_p$-*regularization problems* (1) *satisfies* $\Vert s^* \Vert _0 <k$ *for* $1\le k \le n$.
2. *If* $\lambda \ge \beta (1)$, *then* $s^*=0$ *is the unique global minimizer of the* $l_p$-*regularization problems* (1).

### *Proof*

Assume that $s^*\ne 0$ is a global minimizer of the $l_p$-regularization problems (1). Let $B=A_T\in {R}^{m\times |T |}$, where $T=\mathrm{support(s^*)}$ and $|T |=\Vert s^* \Vert _0$ is the cardinality of the set *T*. Therefore, according to the first order necessary condition, $s^*$ must satisfy

19 $$\begin{aligned} 2B^T(Bs^*_T-b)+\lambda p(|s^*_T |^{p-2}\cdot (s^*_T))=0, \end{aligned}$$

which shows $As^*-b=Bs^*_T-b\ne 0$. Hence, we have

20 $$\begin{aligned} f_\lambda (s^*)=\Vert As^*-b \Vert ^2+\lambda \Vert s^* \Vert _p^p> \lambda \sum \limits _{i\in T} |s^*_i |^p. \end{aligned}$$

By Theorem 3, we can follow that

$$\begin{aligned} |s^*_i |\ge (\lambda \mu (1-p))^{1/(2-p)}, ~ i \in T. \end{aligned}$$

Therefore, we have

21 $$\begin{aligned} f_\lambda (s^*)> \lambda |T | (\lambda \mu (1-p))^{p/(2-p)}. \end{aligned}$$

In the following, we will discuss different cases:

1. Assume that $\lambda \ge \beta (k)$, we shall prove it through apagoge. If $\Vert s^* \Vert _0\ge k \ge 1$, then by (3.11) and the definition of $\beta (k)$ in (3.8), we have
  $$\begin{aligned}\begin{array}{ll} f_\lambda (s^*) &> \lambda |T | (\lambda \mu (1-p))^{p/(2-p)} = k \lambda ^{2/(2-p)} (\mu (1-p))^{p/(2-p)} \\ &\ge k k^{-1} \Vert b \Vert ^2 \\ &= \Vert b \Vert ^2=f_\lambda (0).\\ \end{array} \end{aligned}$$
  This is in contradiction with that $s^*$ is a global minimizer of (1). Therefore, we have $\Vert s^* \Vert _0< k$.
2. Assume that $\lambda \ge \beta (1)$, we shall prove it through apagoge. If $s^* \ne 0$, then there exists $i_0$ satisfying $s^*_{i_0}\ne 0$ and
  $$\begin{aligned} f_\lambda (s^*)=\Vert As^*-b \Vert ^2+\lambda \Vert s^* \Vert ^p_p> \lambda |s^*_{i_0} |^p \ge \lambda (\lambda \mu (1-p))^{p/(2-p)}\ge \Vert b \Vert ^2=f_p(0). \end{aligned}$$

This is in contradiction with that $s^*$ is a global minimizer of (1). Therefore, $s^*=0$ must be the unique global minimizer of (1). $\square$

## Iterative thresholding algorithm and its convergence

By the thresholding representation formula (16), an iterative thresholding formula of the problem (1) can be presented in the following: initilized $s^0\in {R}^n$,

22 $$\begin{aligned} s^{k+1}=H_{\lambda \mu }(s^k+\mu A^T (b-As^k)), \end{aligned}$$

where

23 $$\begin{aligned} h_{\lambda \mu }(r):=\left\{ \begin{array}{lll} h_{\lambda \mu , p}(r) , &\quad r>r^*\\ 0, &\quad -r^* \le r\le r^*\\ -h_{\lambda \mu , p}(-r) , & \quad r<-r^*\\ \end{array} \right. \end{aligned}$$

When $|r| =r^*$, the adjustment here is, we only select $h_{\lambda \mu }(r)= 0$.

Firstly, some important lemmas are given in the following.

### **Lemma 4**

*Let* $0<\mu <\Vert A \Vert ^{-2}$ *and* $\{s^k \}$ *be the sequence produced by the algorithm* (22), *then we can follow that the sequences* $\{ (f_\lambda (s^k))_k \}$ *and* $\{ (f_\mu (s^{k+1}, s^k))_k \}$ *are non-increasing*.

### *Proof*

For $0<\mu <\Vert A \Vert ^{-2}$, we have

$$\begin{aligned} \Vert s^{k+1}-s^k \Vert _2^2-\mu \Vert As^{k+1}-As^{k} \Vert _2^2\ge 0. \end{aligned}$$

Hence,

$$\begin{aligned} \begin{array}{ll} f_{\lambda }(s^{k+1})&\le \mu ^{-1}(\mu f_{\lambda }(s^{k+1})+\Vert s^{k+1}-s^k \Vert _2^2-\mu \Vert As^{k+1}-As^{k} \Vert _2^2) \\ &=\mu ^{-1} f_{\mu }(s^{k+1}, s^k) \\ &\le \mu ^{-1} f_{\mu }(s^{k}, s^k) \\ &=f_{\lambda }(s^{k}) \\ &\le \mu ^{-1}(\mu f_{\lambda }(s^{k})+\Vert s^{k}-s^{k-1} \Vert _2^2-\mu \Vert As^{k}-As^{k-1} \Vert _2^2) \\ &=\mu ^{-1} f_{\mu }(s^{k}, s^{k-1}). \\ \end{array} \end{aligned}$$

The first equality can be followed from the definition of $f_\mu (s, z)$. The second inequality is because that the $s^{k+1}$ is the minimizer of $f_\mu (s, s^k)$. $\square$

This lemma demonstrate that, from iteration to iteration, the objective function $f_\lambda (s)$ does not increase, moreover, using the proposed algorithm does not lead to worse results than not using the proposed algorithm. The algorithm (22) does not have a unique fixed point, therefore it is very important to analyze the fixed points in detail.

### **Lemma 5**

*Let* $\Gamma _0=\{i:~s^*_i=0 \}$ *and* $\Gamma _1=\{i:~|s^*_i|>(\lambda \mu (1-p) )^{1/(2-p)} \}$. *The point* $s^*$ *is a fixed point for the algorithm* (18) *if and only if*

$$\begin{aligned} |A_i^T (b-As^*)|&\le \frac{2-p}{2} \lambda ^{1/(2-p)} [\mu (1-p) ]^{(p-1)/(2-p)}, \quad \mathrm{if} \,\, i\in \Gamma _0, \\ s^*_i&=h_{\lambda \mu , p}(s^*_i+\mu A_i^T (b-As^*)), \quad \mathrm{if} \,\, i\in \Gamma _1. \end{aligned}$$

### *Proof*

A fixed point of the algorithm (22) is any $s^*$ satisfying $s^{*}=H_{\lambda \mu }(s^*+\mu A^T (b-As^*))$, i.e., $s^{*}_i=h_{\lambda \mu }(s^*_i+\mu A_i^T (b-As^*))$. If $i\in \Gamma _0$, the equality holds when and only when $|\mu A_i^T (b-As^*)|\le \frac{2-p}{2(1-p)} [\lambda \mu (1-p) ]^{1/(2-p)}$, i.e., $|A_i^T (b-As^*)|\le \frac{2-p}{2} \lambda ^{1/(2-p)} [\mu (1-p) ]^{(p-1)/(2-p)}$. Similarly, $i\in \Gamma _1$ when and only when $s^*_i=h_{\lambda \mu , p}(s^*_i+\mu A_i^T (b-As^*))$. $\square$

The following lemma demonstrate that the sequence $\{s^k \}$ produced by the algorithm (22) is asymptotically regular, i.e., $\lim _{k\rightarrow \infty } \Vert s^{k+1} -s^k \Vert _2=0$.

### **Lemma 6**

*If* $f_\lambda (s^0)<\infty$, $0<\mu < \Vert A \Vert ^{-2}$ *and assume that* $\{s^k \}$ *be the sequence produced by the algorithm* (22), $\forall \epsilon >0,~\exists K$ *satisfying* $\forall k>K,~\Vert s^{k+1}-s^k \Vert _2^2\le \epsilon$.

### *Proof*

We prove the convergence of $\sum \limits _{k=0}^{K} \Vert s^{k+1}-s^k \Vert _2^2$, which implies the lemma. First of all, we prove that $\sum \limits _{k=0}^{K} \Vert s^{k+1}-s^k \Vert _2^2$ is monotonically increasing. We can follow monotonicity from

$$\begin{aligned} \begin{array}{lll} \sum \limits _{k=0}^{K} \Vert s^{k+1}-s^k \Vert _2^2&=&\sum \limits _{k=0}^{K-1} \Vert s^{k+1}-s^k \Vert _2^2+\Vert s^{K+1}-s^K \Vert _2^2\\ &\ge & \sum \limits _{k=0}^{K-1} \Vert s^{k+1}-s^k \Vert _2^2. \end{array} \end{aligned}$$

Then, we will show the boundness of $\sum\nolimits _{k=0}^{K} \Vert s^{k+1}-s^k \Vert _2^2$. For $0<\mu < \Vert A \Vert ^{-2}$, we have $0<\delta :=1-\mu \Vert A \Vert ^{2} <1$ and

$$\begin{aligned} \Vert s^{k+1}-s^k \Vert _2^2\le \delta ^{-1}(\Vert s^{k+1}-s^k \Vert _2^2-\mu \Vert As^{k+1}-As^{k} \Vert _2^2). \end{aligned}$$

Therefore,

$$\begin{aligned} \begin{array}{ll} \sum \limits _{k=0}^{K} \Vert s^{k+1}-s^k \Vert _2^2&\le \delta ^{-1} \sum \limits _{k=0}^{K} (\Vert s^{k+1}-s^k \Vert _2^2-\mu \Vert As^{k+1}-As^k \Vert _2^2) \\ &\le \delta ^{-1} \sum \limits _{k=0}^{K} \mu (f_\lambda (s^k)-f_\lambda (s^{k+1})) \\ &=\mu \delta ^{-1} (f_\lambda (s^0)-f_\lambda (s^{K+1})) \\ &\le \mu \delta ^{-1} f_\lambda (s^0)< \infty . \\ \end{array} \end{aligned}$$

The second inequality can be followed from the proof of Lemma 4 and the last inequality can be taken from $f_\lambda (s^0)<\infty$. $\square$

In the following, we present an very important property of the algorithm, i.e., any accumulation point of the sequence $\{s^k \}$ is a fixed point of the algorithm (22). Therefore, we have the following theorem and conclusion.

### **Theorem 5**

*If* $f_\lambda (s^0)<\infty$ *and* $0<\mu < \Vert A \Vert ^{-2}$, *then we have the following conclusion: any accumulation point of the sequence* $\{s^k \}$ *produced by the algorithm* (22) *is a fixed point of* (22).

### *Proof*

In Lemma 6, we take $\epsilon <\lambda$. If $|s^k_i |>(\lambda \mu (1-p) )^{1/(2-p)}$ and $s^{k+1}_i=0$, then we have $\Vert s^{k+1}-s^k \Vert _2^2\ge \lambda$, by Lemma 6 which is impossible for $k>K$ for some *K*. Therefore, for large *K*, the set of zero and non-zero coefficients will not change and $|s^k_i|>(\lambda \mu (1-p) )^{1/(2-p)}, ~\forall i \in \Gamma _1, ~k>K$. Assume that $\{s^{k_j} \}$ be a convergent subsequence and $s^*$ be its limit point, i.e.,

24 $$\begin{aligned} s^{k_j}\rightarrow s^*, \quad ~\mathrm{~as~}~k_j\rightarrow +\infty . \end{aligned}$$

By the limitation (24) and Lemma 6, we have

$$\begin{aligned} \Vert s^{k_j+1}-s^* \Vert _2\le \Vert s^{k_j+1}-s^{k_j} \Vert _2+\Vert s^{k_j}-s^* \Vert _2\rightarrow 0, \quad \mathrm{as} \,\, k_j\rightarrow +\infty , \end{aligned}$$

which implies that the sequence $\{s^{k_j+1} \}$ is also convergent to $s^*$. Note that $s^{k_j+1}=H_{\lambda \mu }(B_\mu (s^{k_j}))$, i.e., $s^{k_j+1}_i=h_{\lambda \mu }(s^{k_j}_i+\mu A_i^T(b-As^{k_j})), \mathrm{~for~all}~i=1, 2, \ldots , n$.

Let $\Gamma _0=\{i:~s^*_i=0 \}$ and $\Gamma _1=\{i:~s^*_i\ne 0 \}$. For $s^{k_j},~k_j>K$ for some *K*, if $i\in \Gamma _0$, then by (23) and (7) we have

$$\begin{aligned} |A^T_i(b-As^{k_j})|\le \frac{2-p}{2} \lambda ^{1/(2-p)} [\mu (1-p) ]^{(p-1)/(2-p)}, \end{aligned}$$

therefore, $|A^T_i(b-As^*)|\le \frac{2-p}{2} \lambda ^{1/(2-p)} [\mu (1-p) ]^{(p-1)/(2-p)}$. Similarly, if $i\in \Gamma _1$, then by (23) and (7) we have

$$\begin{aligned} s^{k_j+1}_i=h_{\lambda \mu }(s^{k_j}_i+\mu A_i^T(b-As^{k_j})), ~|s^{k_j}_i+\mu A_i^T(b-As^{k_j}) |> r^*, \end{aligned}$$

where $r^*:=\frac{2-p}{2(1-p)} [\lambda \mu (1-p) ]^{1/(2-p)}$. By Proposition 2, we can follow that the function $h_{\lambda \mu , p}()$ is continuous over $(r^*, +\infty )$ and $( -\infty , r^*)$. Therefore, we follow that $s^*_i=h_{\lambda \mu , p}(s^*_i+\mu A^T_i(b-As^*))$. By Lemma 5, $s^*$ is a fixed point of (22). $\square$

## Numerical experiments

Now we report numerical results to compare the performance of Iterative thresholding algorithm (ITA) ($p=0.5$) for solving (1) (Signal reconstruction) with LASSO to find sparse solutions. The computational test was conducted on a Intel(R) Core(TM)2 Duo CPU E 8400 @3.00GHZ Dell desktop computer with 2.0GHz of memory with using Matlab R2010A.

Consider a real-valued, finite-length signal $x \in R^n$. Suppose *x* is T-sparse, that is, only T of the signal coefficients are nonzero and the others are zero. We use the following Matlab code to generate the original signal, a matrix A and a vector b.

$$\begin{aligned} x_{or}& = zeros(n,1); q = randperm(n); x_{or}(q(1:T)) = 2*randn(T,1);\\ A& = randn(m,n); A = orth(A')'; b= A*x_{or} ; \end{aligned}$$

The computational results for this experiment are displayed in Table 1.

**Table 1:** Comparison of ITA and LASSO algorithm

Problems			LASSO		ITA	
*n*	*T*	*m*	Time	Error	Time	Error
800	60	150	0.572	4.16e−4	0.375	1.15e−4
800	80	180	0.461	3.58e−4	0.252	1.06e−4
2000	160	300	0.853	5.75e−4	0.516	1.62e−4
2000	200	500	0.853	5.86e−4	0.553	1.73e−4

From Table 1 we find that ITA has smaller prediction accuracy than LASSO in shorter time.

## Conclusion

In this paper, an exact lower bound for the absolute value of nonzero entries in each global optimum solution of the problem (1) is established. And the necessary condition for global optimum solutions of the $l_p$-regularization problems is derived, i.e., the global optimum solutions are the fixed points of a vector thresholding operator. In addition, we have derived a sufficient condition on the selection of $\lambda$ for the desired sparsity of global minimizers of the problem (1) with the given (*A*, *b*, *p*). Finally, an iterative thresholding algorithm is designed for solving the $l_p$-regularization problems, and the convergence of algorithm is proved.
